# Association of preceding antithrombotic therapy in atrial fibrillation patients with ischaemic stroke, intracranial haemorrhage, or gastrointestinal bleed and mortality

**DOI:** 10.1093/ehjcvp/pvz063

**Published:** 2019-10-26

**Authors:** Joris J Komen, Tomas Forslund, Aukje K Mantel-Teeuwisse, Olaf H Klungel, Mia von Euler, Frieder Braunschweig, Håkan Wallén, Paul Hjemdahl

**Affiliations:** 1 Division of Pharmacoepidemiology and Clinical Pharmacology, Utrecht Institute for Pharmaceutical Sciences, Utrecht University, the Netherlands; 2 Stockholm County Council, Department of Healthcare Development, Sweden; 3 Department of Medicine Solna, Clinical Epidemiology Unit/Clinical Pharmacology, Karolinska Institutet, Karolinska University Hospital, Stockholm, Sweden; 4 Department of Clinical Science and Education, Karolinska Institutet Stroke Research Network at Södersjukhuset, Stockholm, Sweden; 5 Department of Medicine Solna, Cardiology Unit, Karolinska Institute, Karolinska University Hospital, Stockholm, Sweden; 6 Department of Clinical Sciences, Division of Cardiovascular Medicine, Karolinska Institutet, Danderyd Hospital, Stockholm, Sweden

**Keywords:** Non-vitamin K oral anticoagulant, Warfarin, Antiplatelet agents, Mortality, Bleeding, Stroke

## Abstract

**Aims:**

To analyse 90-day mortality in atrial fibrillation (AF) patients after a stroke or a severe bleed and assess associations with the type of antithrombotic treatment at the event.

**Methods and results:**

From the Stockholm Healthcare database, we selected 6017 patients with a known history of AF who were diagnosed with ischaemic stroke, 3006 with intracranial haemorrhage, and 4291 with a severe gastrointestinal bleed (GIB). The 90-day mortality rates were 25.1% after ischaemic stroke, 31.6% after intracranial haemorrhage, and 16.2% after severe GIB. We used Cox regression and propensity score-matched analyses to test the association between antithrombotic treatment at the event and 90-day mortality. After intracranial haemorrhage, there was a significantly higher mortality rate in warfarin compared to non-vitamin K oral anticoagulant (NOAC)-treated patients [adjusted hazard ratio (aHR) 1.36, 95% confidence interval (CI) 1.04–1.78]. After an ischaemic stroke and a severe GIB, patients receiving antiplatelets or no antithrombotic treatment had significantly higher mortality rates compared to patients on NOACs, but there was no difference comparing warfarin to NOACs (aHR 0.84, CI 0.63–1.12 after ischaemic stroke, aHR 0.91, CI 0.66–1.25 after severe GIB). Propensity score-matched analysis yielded similar results.

**Conclusion:**

Mortality rates were high in AF patients suffering from an ischaemic stroke, an intracranial haemorrhage, or a severe GIB. NOAC treatment was associated with a lower 90-day mortality after intracranial haemorrhage than warfarin.

## Introduction

Non-vitamin K oral anticoagulants (NOACs) have been shown to be at least as safe and efficacious as warfarin,^1^ and superior to aspirin in preventing stroke in patients with atrial fibrillation (AF).^2^ In particular, NOACs markedly reduce the risk for intracranial haemorrhage compared to warfarin. Overall, oral anticoagulant (OAC) and aspirin treatment increase the risks of bleeding similarly,^2^^,^^3^ but misconceptions about the safety of aspirin have most likely contributed to undertreatment with OACs and overtreatment with aspirin in AF patients.^4^ In line with the emerging evidence, the recent guidelines advocate increasing OAC treatment, preferably with NOACs.^5^^,^^6^

Previous studies have found associations between antithrombotic treatment at the time of an ischaemic stroke or an intracranial haemorrhage and in-hospital mortality.^7^^,^^8^ Work by Hylek *et al*.^9^ showed that mortality in the 30 days post-discharge is as large as the in-hospital mortality in AF patients suffering from an ischaemic stroke. Studies with a longer follow-up, capturing both in-hospital and early out-of-hospital mortality after an ischaemic stroke or intracranial haemorrhage in the NOAC era have not been reported. Studies describing the outcomes of AF patients suffering from a severe gastrointestinal bleed (GIB) even appear to be lacking.

The aims of the current study were therefore to analyse the 90-day mortality in patients suffering from an ischaemic stroke, an intracranial haemorrhage, or a severe GIB, and to assess if this is associated with the type of antithrombotic treatment at the time of the event.

## Materials and methods

### Patient selection

For this population-based cohort study, we used the Stockholm Healthcare Database (VAL database, see Supplementary material online, *eMethods*). From this database, we created three cohorts: one with patients with ischaemic stroke, one with patients with intracranial haemorrhage, and one with patients with a severe GIB, registered between July 2011 and June 2018. All patients had a prior diagnosis for AF (I48) (see Supplementary material online, *eTable 1* for ICD-10 codes). Patients could be included in more than one cohort. We only included diagnoses recorded in a hospital care setting requiring acute somatic care. For ischaemic strokes we only included diagnoses registered as primary or secondary diagnosis in inpatient care. For intracranial haemorrhage and severe GIBs the diagnoses could be in any position and could be recorded in inpatient care or at an acute hospital-based emergency visit.^10^ Validation studies in the same database have shown a positive predictive value of 98.6% for ischaemic stroke, 97.7% for intracranial haemorrhage, and 98.1% for GIBs.^10^^,^^11^

### Follow-up, outcome, and censoring

We defined the date of the qualifying event as the index date, and followed patients for a maximum of 90 days. The outcome of interest during follow-up was all cause mortality, registered at Statistics Sweden. Patients were censored if they moved out from the region during follow-up.

### Baseline treatment assessment

Baseline treatment at the time of the bleed or stroke could be any of the following four classes: NOAC, warfarin, antiplatelet, or no treatment (see Supplementary material online, *eTable 1* for ATC codes). NOAC treatment included all four NOACs (dabigatran, rivaroxaban, apixaban, and edoxaban), and the antiplatelet treatment was low-dose aspirin and/or P2Y_12_ antagonist treatment (clopidogrel, ticagrelor, and prasugrel). Baseline treatment was defined based on prescriptions that were theoretically available at the time of the event (see Supplementary material online, *eMethods*).

### Baseline comedication and comorbidity definition

We defined baseline comedication as prescriptions claimed during 6 months prior to inclusion, i.e. the bleed or stroke. We searched for prescriptions for diuretics, beta-blockers, calcium channel blockers, renin angiotensin aldosterone system (RAAS) inhibitors, statins, oral antidiabetic drugs, insulins, antidepressants, digoxin, rhythm control drugs, non-steroidal antiinflammatory drugs (NSAIDs), corticosteroids, and proton pump inhibitors (see Supplementary material online, *eTable 1* for ATC codes).

We defined baseline comorbidity as all recorded diagnoses in the 5 years prior to inclusion (see Supplementary material online, *eTable 1* for ICD codes). We assessed the comorbidities of the Charlson Comorbidity Index, the CHA_2_DS_2_-VASc score, and the modified HAS-BLED score.^12–14^ Comorbidities that occurred in more than one score were counted only once. The Charlson Comorbidity Index includes the following: myocardial infarction, heart failure, peripheral vascular disease, cerebral vascular disease, dementia, chronic obstructive pulmonary disease, peptic ulcer, rheumatoid arthritis, mild liver disease, uncomplicated diabetes, connective tissue disease, renal disease, complicated diabetes, cancer, moderate to severe liver disease, metastatic carcinoma, and HIV. For the CHA_2_DS_2_-VASc score, we also assessed hypertension and previous stroke, transient ischaemic attack (TIA), or embolism. For the modified HAS-BLED score, we assessed anaemia, alcoholism, and prior bleeds; PK(INR) values were not available.

### Statistical analysis

We used basic descriptive statistics to present baseline characteristics of the three cohorts and to calculate the crude 90-day mortality rates. We used a Cox proportional hazards model to calculate adjusted hazard ratios (aHRs), correcting for potential confounders.^15^ In the Cox proportional hazards model, we adjusted for age, sex, the individual components of the Charlson Comorbidity Index, the CHA_2_DS_2_-VASc score, and the modified HAS-BLED score, for baseline medication as described above, and for the year of inclusion. We created models for several comparisons, to assess underlying relationships. We compared NOACs with warfarin, antiplatelets, and no treatment. We tested the proportional hazards assumptions of the Cox regression with Schoenfeld residuals.^16^ In addition, we performed propensity score-matched analyses (see Supplementary material online, *eMethods*).

Data extraction was performed using SAS EG 7.1 (SAS Institute Inc., Cary, NC, USA), all statistical analyses were performed with statistical software R version 3.4.2 and RStudio Desktop version 1.1.463. The statistical packages ‘survival’ and ‘MatchIt’ were used for the survival analyses and the propensity score matching, respectively.^17^^,^^18^

### Sensitivity analysis

We performed several sensitivity analyses; an array-approach sensitivity analysis for unmeasured confounding, an asymmetric trimmed propensity score-matched analysis, an analysis with different exposure windows, an analysis including intracranial haemorrhages and GIBs only as primary inpatient diagnosis, and an analysis which excluded all patients receiving concomitant antiplatelet therapy (see Supplementary material online, *eMethods*).^19^^,^^20^

## Results

### Patient characteristics

A total of 105 313 patients in the Stockholm region were diagnosed with AF in the VAL database during the period of inclusion. Among these patients, 6017 had an ischaemic stroke, 3006 an intracranial haemorrhage, and 4291 a severe GIB after their diagnosis of AF. Patients suffering from an ischaemic stroke were the oldest with a mean age of 81.6 years. The mean ages were 80.2 years for intracranial haemorrhage and 78.7 years for severe GIB.

Among the patients with ischaemic stroke, 454 (7.5%) were using NOACs, 1229 (20.4%) were using warfarin, 2026 (33.7%) were using antiplatelets, and 2308 (38.4%) had not claimed any antithrombotic treatment (see *Table 1*). The proportion of ischaemic stroke patients without OAC treatment decreased from 80.2% in 2011 to 58.8% in 2018. Patients receiving antiplatelets were older and had higher risk scores than the other three groups, which were comparable.

**Table 1:** Baseline characteristics of patients included after ischaemic stroke, intracranial haemorrhage, and severe gastrointestinal bleed

Baseline characteristics	NOAC	Warfarin	Antiplatelet	No treatment
Ischaemic stroke (*n* = 6017)				
Number of patients	454	1229	2026	2308
Female gender, *n* (%)	237 (52.2)	577 (46.9)	1149 (56.7)	1238 (53.6)
Mean age (years) (SD)	79.25 (9.35)	80.62 (8.45)	83.89 (9.24)	80.64 (10.60)
Mean Charlson Comorbidity Index (SD)	5.59 (2.35)	5.92 (2.40)	6.18 (2.39)	5.75 (2.59)
Mean CHA_2_DS_2_-VASc score (SD)	4.43 (1.68)	4.66 (1.64)	4.80 (1.69)	4.23 (1.84)
Mean HAS-BLED score (SD)	2.32 (0.88)	2.29 (0.84)	2.36 (0.95)	2.25 (1.06)
Concomitant antiplatelet,^a^ *n* (%)	37 (8.1)	107 (8.7)	49 (2.4)	NA
Mean treatment duration (years) (SD)^b^	1.2 (1.2)	2.9 (2.0)	2.7 (1.8)	0.8 (1.0)
Intracranial haemorrhage (*n* = 3006)				
Number of patients	311	1028	595	1072
Female gender, *n* (%)	132 (42.4)	415 (40.4)	275 (46.2)	442 (41.2)
Mean age (years) (SD)	80.02 (9.12)	79.62 (8.75)	83.02 (9.32)	79.32 (10.92)
Mean Charlson Comorbidity Index (SD)	5.83 (2.53)	5.80 (2.46)	6.52 (2.58)	5.93 (2.83)
Mean CHA_2_DS_2_-VASc score (SD)	4.33 (1.71)	4.31 (1.64)	4.75 (1.64)	4.07 (1.83)
Mean HAS-BLED score (SD)	2.35 (0.91)	2.26 (0.85)	2.51 (0.98)	2.36 (1.02)
Concomitant antiplatelet,^a^ *n* (%)	5 (1.6)	25 (2.4)	12 (2.0)	NA
Mean treatment duration (years) (SD)^b^	1.4 (1.3)	3.1 (2.1)	2.9 (2.0)	0.6 (0.7)
Severe gastrointestinal bleed (*n* = 4291)				
Number of patients	652	1293	893	1453
Female gender, *n* (%)	300 (46.0)	526 (40.7)	412 (46.1)	607 (41.8)
Mean age (years) (SD)	77.84 (9.36)	78.39 (9.60)	81.59 (10.40)	77.68 (11.43)
Mean Charlson Comorbidity Index (SD)	5.77 (2.63)	6.09 (2.65)	6.61 (2.68)	6.29 (3.09)
Mean CHA_2_DS_2_-VASc score (SD)	4.21 (1.81)	4.26 (1.65)	4.58 (1.74)	3.93 (1.86)
Mean HAS-BLED score (SD)	2.25 (0.93)	2.26 (0.92)	2.41 (1.02)	2.34 (1.17)
Concomitant antiplatelet,^a^ *n* (%)	41 (7.8)	129 (8.1)	54 (2.5)	NA
Mean treatment duration (years) (SD)^b^	1.3 (1.2)	2.9 (2.1)	3.0 (2.0)	0.8 (1.0)

Complete baseline tables with all comedication and comorbidities can be found in Supplementary material online, *eTable 2a–c*.

NA, not applicable; NOAC, non-vitamin K oral anticoagulant; SD, standard deviation.

a Concomitant antiplatelet is either NOAC + antiplatelet, warfarin + antiplatelet, or double antiplatelet therapy.

b For the no treatment group, this is the mean number of years since a last prescription, only among patients who ever received any antithrombotic treatment.

Among the patients with intracranial haemorrhage, 311 (10.3%) were using NOACs, 1028 (34.2%) were using warfarin, 595 (19.8%) were using antiplatelets, and 1072 (35.7%) had not claimed any antithrombotic treatment (see *Table 1*). Among the patients with severe GIB, 652 (15.2%) were using NOACs, 1293 (30.1%) warfarin, 893 (20.8%) antiplatelets, and 1453 (33.9%) no treatment (see *Table 1*). Again, patients on antiplatelets were older and had higher risk scores, but patients on NOACs, warfarin, and no treatment were comparable also in the two cohorts with bleeds.

The proportion of NOAC patients treated with a low dose was 48.0% in the ischaemic stroke group, 43.7% in the intracranial haemorrhage group, and 39.0% in the severe GIB group. The proportion of patients receiving combination therapy (i.e. OAC with antiplatelet or double antiplatelet therapy) was small; below 10% in all groups (see *Table 1*).

After propensity score matching, all covariates had a standardized mean difference below 0.1, indicating successful matching in all three cohorts (see Supplementary material online, *eTable 3a–c*).

### Mortality

The 90-day mortality was 25.1% after an ischaemic stroke, 31.6% after an intracranial haemorrhage, and 16.2% after a severe GIB, regardless of antithrombotic treatment at the time of the event).

### Ischaemic stroke

Both NOAC- and warfarin-treated patients had 90-day mortalities of 17.6%. For antiplatelet-treated patients this was 29.8% and for patients without treatment 26.3% (see *Table 2*). After adjustment for confounders, patients receiving antiplatelets or no treatment had significantly higher mortality rates compared to patients on NOAC treatment [antiplatelet vs. NOAC, aHR 1.57, 95% confidence interval (CI) 1.20–2.04; no treatment vs. NOAC, aHR 1.47, CI 1.15–1.88, see *Table 2*]. There was no statistically significant difference in mortality rates between warfarin- and NOAC-treated patients, either in the adjusted Cox regression or in the propensity score-matched cohort (see *Figure 1A*).

**Figure 1:**
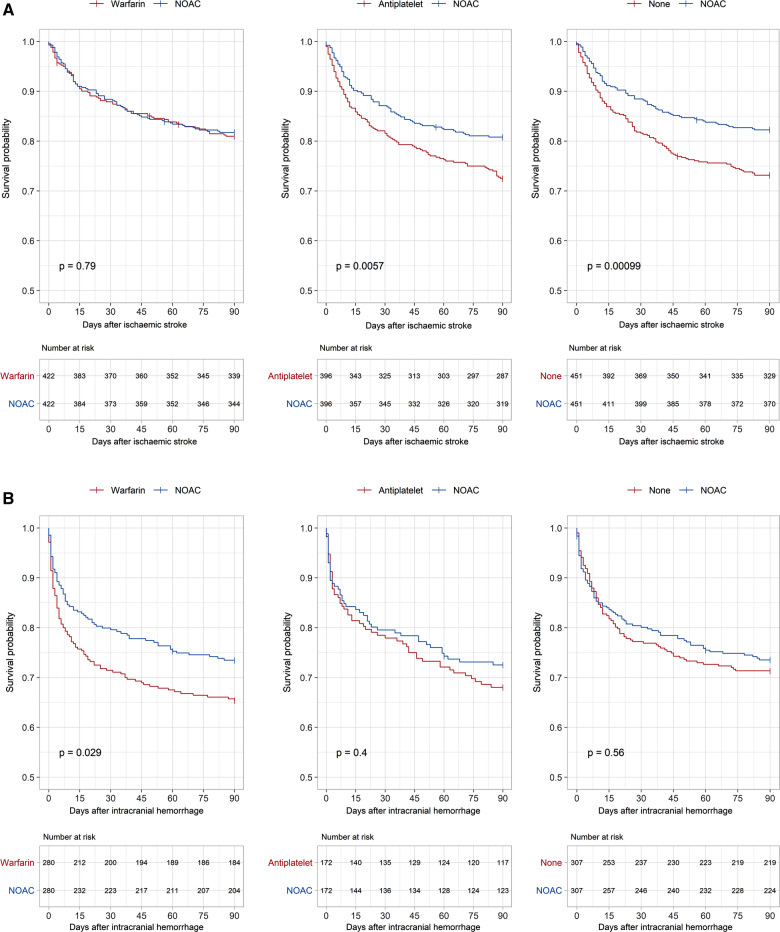
(*A*) 90-Day mortality after ischaemic stroke. Kaplan–Meier curves and *P*-values from the log-rank test in the propensity score-matched cohorts after ischaemic stroke. (*B*) 90-Day mortality after intracranial haemorrhage. Kaplan–Meier curves and *P*-values from the log-rank test in the propensity score-matched cohorts after intracranial haemorrhage. (*C*) 90-Day mortality after severe gastrointestinal bleed. Kaplan–Meier curves and *P*-values from the log rank test in the propensity score-matched cohorts after severe gastrointestinal bleed. NOAC, non-vitamin K oral anticoagulant.

**Figure 1:**
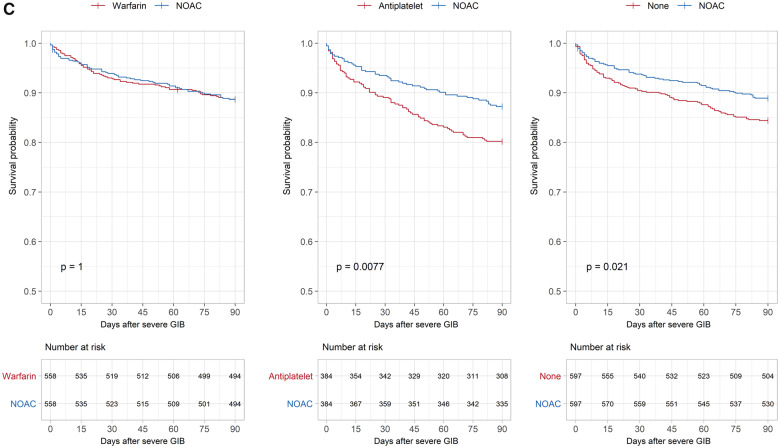
Continued.

**Table 2:** 90-Day mortality rates in the different treatment groups after ischaemic stroke, intracranial haemorrhage, and severe gastrointestinal bleed

	NOAC	Warfarin	Antiplatelet	No treatment
Ischaemic stroke				
90-Day mortality, *n* (%)	80 (17.6)	216 (17.6)	604 (29.8)	608 (26.3)
Unadjusted HR (CI)	Reference	1.00 (0.77–1.29)	1.84 (1.45–2.32)	1.58 (1.25–2.00)
Adjusted HR (CI)	Reference	0.84 (0.63–1.12)	1.57 (1.20–2.04)	1.47 (1.15–1.88)
Intracranial haemorrhage				
90-Day mortality, *n* (%)	82 (26.4)	333 (32.4)	220 (37.0)	315 (29.4)
Unadjusted HR (CI)	Reference	1.30 (1.02–1.66)	1.49 (1.16–1.92)	1.13 (0.88–1.44)
Adjusted HR (CI)	Reference	1.36 (1.04–1.78)	1.16 (0.84–1.61)	1.02 (0.78–1.34)
Severe gastrointestinal bleed				
90-Day mortality, *n* (%)	71 (10.9)	147 (11.4)	194 (21.7)	284 (19.5)
Unadjusted HR (CI)	Reference	1.05 (0.79–1.39)	2.13 (1.62–2.80)	1.89 (1.45–2.45)
Adjusted HR (CI)	Reference	0.91 (0.66–1.25)	1.56 (1.13–2.16)	1.51 (1.13–2.01)

HRs from the unadjusted and adjusted Cox regression models, adjusted for age, sex, the individual components of the Charlson Comorbidity Index, the CHA_2_DS_2_-VASc score, and the modified HAS-BLED score, for baseline medication, and for the year of inclusion.

CI, 95% confidence interval; HR, hazard ratio; NOAC, non-vitamin K oral anticoagulant.

### Intracranial haemorrhage

Among patients with an intracranial haemorrhage, the lowest 90-day mortality was found in NOAC-treated patients (26.4%), and the highest in patients receiving antiplatelets (37.0%). After adjusting for confounders, there was a significantly increased risk of dying among warfarin compared to NOAC-treated patients (aHR 1.36, CI 1.04–1.78). In patients on antiplatelets and in patients without antithrombotic treatment, there were no significant differences in mortality risk compared to NOAC-treated patients. The log-rank test in the propensity score-matched cohorts yielded similar results (see *Figure 1B*).

### Gastrointestinal bleeds

The lowest 90-day mortality was again found in NOAC-treated patients (10.9%), while the mortality in antiplatelet-treated patients was twice as high (21.7%). After adjustment for confounders, patients receiving antiplatelets or no treatment had significantly higher mortalities compared to NOACs (antiplatelet vs. NOAC, aHR 1.56, CI 1.13–2.16; no treatment vs. NOAC, aHR 1.51, CI 1.13–2.01). There was no statistically significant difference between NOAC- and warfarin-treated patients (aHR 0.96, CI 0.72–1.29). The log-rank test in the propensity score-matched cohorts again yielded similar results (see *Figure 1C*).

### Sensitivity analyses

The sensitivity analyses showed that the results were robust, i.e. independent of exposure and outcome definitions, and unlikely explained by residual confounding (see Supplementary material online, *eResults Sensitivity Analyses*).

## Discussion

In this observational study covering a complete healthcare setting, we found high 90-day mortalities in AF patients suffering from an ischaemic stroke, an intracranial haemorrhage, or a severe GIB, requiring acute hospital-based emergency care or inpatient care. The 90-day mortalities were 25.1%, 31.6%, and 16.2%, respectively, regardless of antithrombotic treatment at the time of the event. A high proportion of AF patients, i.e. approximately 2 out of 3 patients, were apparently without OAC treatment at the time of an ischaemic stroke.

After an intracranial haemorrhage, the mortality was significantly lower among patients treated with a NOAC compared to those treated with warfarin, both in the adjusted Cox regression and in a propensity score-matched analysis. A possible explanation could be that intracranial haemorrhages occurring during warfarin treatment are associated with larger expansion of haematoma volumes than those observed during NOAC treatment.^21^ Warfarin acts on several coagulation factors and the brain is rich in subendothelial tissue factor which can generate thrombin locally; warfarin may thus counteract locally formed thrombin more effectively than the NOACs and cause more protracted bleeding.^21^ The four pivotal clinical trials showed lower risks for intracranial haemorrhage with NOACs compared to warfarin.^1^ Our study adds that patients also had a better survival after an intracranial haemorrhage when treated with a NOAC. Although intracranial haemorrhage is a rare complication, the favourable effects of NOACs on the risk of intracranial haemorrhage and the survival after intracranial haemorrhage may add to the improved overall survival that is suggested in the clinical trials.

For ischaemic stroke and severe GIB, the mortality rates were similar in patients on warfarin and NOAC treatment, while mortality rates were significantly higher in patients receiving antiplatelets or no antithrombotic therapy. The lower mortality rates after ischaemic strokes occurring during OAC treatment compared to non-OAC treatment could potentially be explained by fewer thrombi from a cardiac source, smaller thrombi, or both.^22^^,^^23^ Lower mortality rates after a severe GIB during NOAC treatment could potentially be explained by less careful follow-up of patients treated with antiplatelets or no treatment and bleeds being discovered later. However, residual confounding might also be part of the explanation.

### Other literature

Our findings are in line with previous publications with shorter follow-up which reported on in-hospital mortality rates only. Xian *et al*.^8^ reported similar in-hospital mortality rates after an ischaemic stroke for NOAC- and warfarin-treated patients, while patients receiving no antithrombotic treatment had higher in-hospital mortality rates. Studies conducted before NOACs were available also showed a lower in-hospital mortality when comparing warfarin with aspirin or no antithrombotic treatment in AF patients suffering from an ischaemic stroke.^9^^,^^24^ In the current study we had no access to PK(INR) measurements while previous work showed that subtherapeutic warfarin is associated with worse outcomes after stroke and intracranial haemorrhage.^7–9^ The difficulty of warfarin dosing is a drawback of the treatment and our results represent clinical practice. However, it has been reported that warfarin treatment is delivered with high quality and excellent time in therapeutic range values in Sweden and Stockholm.^25^^,^^26^

Inohara *et al*.^7^ found an increased in-hospital mortality after an intracranial haemorrhage in patients with warfarin compared to NOAC treatment, in agreement with our findings. However, when comparing OACs vs. no OACs, they found a reduced in-hospital mortality in patients without OAC treatment, while we found no such association. An explanation could be that we focused solely on AF patients, while Inohara *et al*. included all patients with an intracranial haemorrhage. As a result, the no OAC population in that study was approximately 10 years younger than the OAC population in our study (68 vs. 78 years of age), and also 12 years younger than our intracranial haemorrhage cohort (68 vs. 80 years of age).

### Clinical implications

We are the first to address mortality after the occurrence of a severe GIB in an AF population, which was 16.2% overall after 90 days. For comparison, previous work in the Stockholm region showed a 1-year mortality rate in all AF patients of 8.4%, and in the elderly AF population (age ≥ 80 years) of 16.0%.^27^ The present findings show that mortality rates in the 90 days after a severe GIB are as high as the 1-year mortality in the elderly AF population. Increased awareness and follow-up of these patients is warranted, especially during the first months after the event. In addition, we found that 72% of AF patients suffering from an ischaemic stroke were not receiving OAC treatment. Even with prolonged exposure windows in the sensitivity analysis, 62% of those patients were not receiving OAC treatment. Not only does OAC treatment reduce the risk for an ischaemic stroke, but mortality rates are also higher in patients without OAC treatment at the time of the ischaemic stroke. Finally, guidelines have recommended NOACs above warfarin for stroke prevention in AF, partly due to the reduced risk for intracranial haemorrhage. The current study adds that intracranial haemorrhages occurring while receiving NOAC treatment were also associated with lower mortality rates.

### Strengths

Our study has several strengths. First, the VAL database contains complete follow-up and healthcare utilization data for all patients in the region, giving a unique opportunity to study clinical practice-based outcomes in patients suffering strokes or serious bleeds. Second, we used different analytical approaches and sensitivity analyses, all yielding similar results and confirming the robustness of our findings. Third, we are the first to address outcomes with a longer follow-up after an event. In comparison, we found 90-day mortalities of 31.6% after an intracranial haemorrhage and 27.0% after an ischaemic stroke, while this was only 24% after intracranial haemorrhage and 8% after stroke in studies assessing only in-hospital mortality.^7^^,^^8^

### Limitations

Our study has some limitations. First, the study relies on pharmacy claims data, so we cannot be sure that the patients actually took the medication at the time of the event. Changing the exposure definition and defining a patient treated in the 180 days after a prescription reduced the proportion of untreated patients, but mortality rates remained unchanged, adding to the robustness of our findings. Second, no information is available on the use of reversing therapies after bleeding. Idarucizumab, a dabigatran antidote, became available during the study period, but only 1.9% of all ICH patients used dabigatran (18% of NOAC patients), and 4.0% of all severe GIB patients used dabigatran (26% of NOAC patients). Andexanet alfa, a factor Xa inhibitor antidote, was not available during the study period. Third, patients who died from the event before reaching the hospital were not captured in our database as causes of death were not available for this study. Furthermore, causes of death were not analysed in the presently identified patients with events since the very low autopsy rates in Sweden most likely result in frequent misclassification.^28^ Fourth, antithrombotic treatment after the event, which may have affected mortality, was not taken into account. Finally, despite the efforts made we cannot rule out residual confounding. However, we found that an unmeasured confounder needed an RR of 2.0 and occurring in 50% of the warfarin patients and only 10% of the NOAC patients to explain the association with mortality after an intracranial haemorrhage. It is unlikely that we, after adjusting for many known risk factors, have missed a confounder or group of confounders that is so strongly associated with mortality and so unevenly distributed. Therefore, the associations we observed are not likely to be explained by residual confounding.

We did not take adequacy of NOAC dose and PK(INR) into account in the current study. However, the study describes a clinical practice-based setting in which inadequacy of dosing and low time in therapeutic ranges (TTRs) are part of everyday treatment with OACs. Therefore, the results of this study give a realistic picture of what mortality rates will look like in clinical practice. We did not study reinitiations of antithrombotic treatment after ischaemic or bleeding events since it is impossible to determine if and when a patient with a drug supply from before the event used that after the event. Data on new prescriptions and claims after the event would be seriously confounded by concealed use. We decided to analyse a relatively short follow-up, so that reinitiation of therapy would be expected to have a limited effect on mortality. However, future studies addressing post-event antithrombotic treatment are of interest and warranted.

## Conclusion

In conclusion, the 90-day mortality was high among AF patients suffering from an ischaemic stroke, an intracranial haemorrhage, or a severe GIB. Treatment at the time of the event was associated with 90-day mortality. After an intracranial haemorrhage, patients had better chances of surviving if they received NOAC treatment before the event as compared to warfarin treatment. After a severe GIB or an ischaemic stroke, patients had lower mortality rates if they had received NOAC treatment compared to no OAC treatment. The results of this study support current guidelines that recommend NOACs as first line treatment in stroke prevention in AF.

## Supplementary material

Supplementary material is available at *European Heart Journal – Cardiovascular Pharmacotherapy* online.

## Supplementary Material

pvz063_Supplementary_Data
