# Supplementary material for: Association of preceding antithrombotic therapy in atrial fibrillation patients with ischaemic stroke, intracranial haemorrhage, or gastrointestinal bleed and mortality
Source: Eur Heart J Cardiovasc Pharmacother. 2019 Oct 26;7(1):3–10. doi: 10.1093/ehjcvp/pvz063 (PMC7811399; doi:10.1093/ehjcvp/pvz063)
Supplement: pvz063_Supplementary_Data [file pvz063_supplementary_data.docx]

**Online-Only Supplement**

**eMethods**

**eResults sensitivity analysis**

**eTables**

**eTable 1:** ICD-10 and ATC codes for inclusion, comorbidities, and medication

**eTable 2a:** Complete baseline characteristics ischemic stroke cohort.

**eTable 2b:** Complete baseline characteristics intracranial hemorrhage cohort.

**eTable 2c:** Complete baseline characteristics severe gastrointestinal bleed cohort.

**eTable 3a:** Baseline characteristics and standardized mean differences of the propensity score matched cohort after ischemic stroke.

**eTable 3b:** Baseline characteristics and standardized mean differences of the propensity score matched cohort after intracranial hemorrhage.

**eTable 3c:** Baseline characteristics and standardized mean differences of the propensity score matched cohort after severe gastrointestinal bleed.

**eTable 4:** Array approach sensitivity analyses for unmeasured confounder.

**eTable 5:** Results of sensitivity analysis with different exposure definition.

**eTable 6:** Results of the sensitivity analysis including only primary diagnosis from primary care.

**eTable 7:** Results of sensitivity analysis excluding all patients with concomitant antiplatelet therapy.

**eFigures**

**eFigure 1:** Kaplan-Meier curves and p-values from the log-rank test in the trimmed propensity score matched cohorts after ischemic stroke.

**eFigure 2:** Kaplan-Meier curves and p-values from the log-rank test in the trimmed propensity score matched cohorts after intracranial hemorrhage

**eFigure 3:** Kaplan-Meier curves and p-values from the log rank test in the trimmed propensity score matched cohorts after severe gastrointestinal bleed.

**eMethods**

**Database**

The Stockholm Healthcare Database (Vårdanalysdatabasen, VAL-database) which contains pseudonymized information on demographics, claimed prescriptions, and medical information for all 2.3 million inhabitants in the Stockholm region ^1^.These individual-level data provide the opportunity to have complete healthcare data for follow-up of all patients in the region.

The medical information in the VAL database comes from both primary and secondary care and is registered with ICD-10 codes. Diagnoses and procedures from secondary care have been available since 1993 and from primary care since 2003. The claimed prescription data in the database contain information on drugs claimed in any pharmacy in Sweden and is registered with ATC codes. Data on claimed prescriptions have been included in the VAL database since July 2010 ^2^. Linkage within the database is done using the Swedish unique personal identifier ^3^.

**Baseline treatment assessment**

We searched for a claimed prescription of any of the treatments that was theoretically available for the patient at the time of the bleed or stroke. For the NOACs and antiplatelets we calculated the end of a prescription by assessing the amount of drug dispensed. For warfarin, we used a 90 day period as the duration for a prescription, given the diversity in warfarin dosing. If the qualifying bleed or stroke was within the duration of the prescription, we allocated the patient to that treatment class.

Baseline exposure to any of the drugs was mutually exclusive, where NOAC or warfarin treatment overruled antiplatelet therapy. Therefore, if a patient had a prescription for both an antiplatelet and a NOAC or warfarin at the time of inclusion, the patient was allocated to the NOAC or warfarin group. However, we assessed the proportion receiving combination therapy at the time of the events. If the patient had a prescription for both a NOAC and warfarin at the time of inclusion, the patient was allocated to the last of the two drugs claimed. If a patient had no treatment available at the time of the bleed or stroke, the patient was considered to have no treatment.

**Propensity score matching**

In addition to the Cox regression, we performed propensity score matched analyses. We thus calculated the probability of receiving NOAC treatment using logistic regression, using the same explanatory variables as in the Cox regression. We matched NOAC to warfarin users, NOAC to antiplatelet users, and NOAC users to non-users on the propensity score. We used a 1:1 nearest neighbor matching method with a greedy matching procedure, using calipers of 0.2 of the standard deviation of the logit of the propensity score. After matching, we created Kaplan Meier curves and performed log rank tests, comparing patient groups that had on average the same distribution of characteristics that were included in the propensity score. Baseline characteristics were compared after the matching, and if all standardized mean differences (SMD) were below 0.1, the matching was considered successful. If not, we would re-estimate the propensity score and re-match the cohorts.

**Sensitivity analyses**

First, to estimate the influence of unmeasured confounding on mortality, we performed sensitivity analyses based on an array approach according to Schneeweiss ^4^. This analysis assesses both how strong the association of the confounder with the outcome must be, and how unequally the confounder must be distributed to fully explain the observed association. We used the analyses for both the weakest and the strongest significant associations.

Second, we conducted another propensity score matched analysis, but now with asymmetric propensity score trimming at cut points corresponding to the 5^th^ and 95^th^ percentiles of the propensity score distribution in the treated and untreated patients, respectively. This approach, as suggested by Stürmer et al, will limit unmeasured confounding since patients in the upper and lower tails of the propensity score distribution are excluded ^5^.

Third, we performed sensitivity analyses in which we assumed a patient was exposed if any treatment was claimed in the 180 days before the event, to account for potential non-compliance.

Fourth, as bleeds recorded in an emergency hospital setting might be less severe than those recorded in an inpatient setting, we have performed an additional analysis where we only included patients with an intracranial hemorrhage or a GIB that was recorded as primary diagnosis in inpatient care.

Fifth, as patients receiving concomitant antiplatelet therapy might have different mortality rates, we performed an analysis where we excluded all patients receiving concomitant antiplatelet therapy.

**eResults sensitivity analyses**

The array approach analyses showed that for the lowest significant association found (aHR: 1.36, warfarin vs NOAC after intracranial hemorrhage) there had to be an unmeasured confounder with a relative risk for mortality of 2.0, occurring 5 times more often in the warfarin group (i.e. 10% in the NOAC group, 50% in the warfarin group) to move the hazard ratio to 1, or an unmeasured confounder with a relative risk for mortality of 3.0 occurring 3 times more often in the warfarin group (see eTable 4). For the strongest association (aHR: 1.57, antiplatelet vs NOAC treatment after ischemic stroke), an unmeasured confounder with a relative risk of 3.0 for mortality had to occur 5 times more often in the antiplatelet group to move the hazard ratio below 1.

The propensity score matched analyses with asymmetric trimming yielded similar results as the main analyses, with all significant results from the main analysis remaining significant and indicating limited residual confounding (eFigure 1a-c).

Considering a patient to be exposed if a drug was claimed 180 days before inclusion yielded similar results as the main analyses (eTable 5).

When including only primary diagnosis from inpatient care, the mortality rates remained similar after intracranial hemorrhages, but increased slightly to 19.1% after gastrointestinal bleeds. The mortality rates in the different treatment groups increased non-differentially, and the associations are similar as in the main analysis (eTable 6).

When excluding all patients receiving concomitant antiplatelet therapy, the mortality rates in the different treatment groups after the different events, remained practically unchanged (eTable 7).

**eReferences**

1. Forslund T, Wettermark B, Wändell P, Euler M von, Hasselström J, Hjemdahl P. Risk scoring and thromboprophylactic treatment of patients with atrial fibrillation with and without access to primary healthcare data: Experience from the Stockholm health care system. *Int J Cardiol* 2013;**170**:208–214.

2. Wettermark B, Hammar N, Fored CM, MichaelFored C, Leimanis A, Otterblad Olausson P, Bergman U, Persson I, Sundström A, Westerholm B, Rosén M. The new Swedish Prescribed Drug Register--opportunities for pharmacoepidemiological research and experience from the first six months. *Pharmacoepidemiol Drug Saf* 2007;**16**:726–735.

3. Ludvigsson JF, Otterblad-Olausson P, Pettersson BU, Ekbom A. The Swedish personal identity number: possibilities and pitfalls in healthcare and medical research. *Eur J Epidemiol* 2009;**24**:659–667.

4. Schneeweiss S. Sensitivity analysis and external adjustment for unmeasured confounders in epidemiologic database studies of therapeutics. *Pharmacoepidemiol Drug Saf* John Wiley & Sons, Ltd; 2006;**15**:291–303.

5. Sturmer T, Rothman KJ, Avorn J, Glynn RJ. Treatment Effects in the Presence of Unmeasured Confounding: Dealing With Observations in the Tails of the Propensity Score Distribution--A Simulation Study. *Am J Epidemiol* Oxford University Press; 2010;**172**:843–854.

**eTable 1: ICD-10 and ATC codes for inclusion, comorbidities, and medication**

| **Diagnosis for inclusion** | **ICD-code beginning with** |
| --- | --- |
| Ischaemic stroke | I63 |
| Intracranial haemorrhage | I60, I61, I62, S064, S065, S066 |
| Gastrointestinal bleed | K25-28 (subcodes 0-2 and 4-6 only), K625, K922 |
| **Baseline comorbidities** | **ICD-code beginning with** |
| Myocardial infarction | I21, I22, I252 |
| Heart failure | I43, I50, I099, I110, I130, I132, I255, I420, I425-429, P290 |
| Peripheral vascular disease | I70, I71, I731, I738, I739, I711, I790, I792, K551, K558, K559, Z958, Z959 |
| Cerebral vascular disease | G45, G46, I60-69, H340 |
| Dementia | F00-03, G30, F051, G311 |
| COPD | J40-47, J60-67, I278, I279, J684, J701, J703 |
| Peptic ulcer | K25-28 |
| Rheumatoid arthritis | M05, M06, M32-34, M315, M351, M353, M360 |
| Mild liver disease | B18, K73, K74, K700, K701, K702, K703, K709, K717, K713, K714, K715, K760, K762, K763, K764, K768, K769, Z944 |
| Uncomplicated diabetes | E100, E101, E106, E108-111, E118, E119, E120, E121, E126, E128-131, E136, E138-141, E146, E148, E149 |
| Connective tissue disease | G81, G82, G041, G114, G801, G802, G830, G831, G832, G833, G834, G839 |
| Renal disease | N18, N19, N052, N053, N054, N055, N056, N057, N250, I120, I131, N032, N033, N034, N035, N036, N037, Z490, Z491, Z492, Z940, Z992 |
| Complicated diabetes | E102, E103, E104, E105, E107, E112, E113, E114, E115, E117, E122, E123, E124, E125, E127, E132, E133, E134, E135, E137, E142, E143, E144, E145, E147 |
| Cancer | C0, C1, C20-26, C30-34, C37-39, C40-43, C45-49, C50-58, C6, C70-76, C81-85, C88, C90-97 |
| Moderate to severe liver disease | K704, K711, K721, K729, K765, K766, K767, I850, I859, I864, I982 |
| Metastatic carcinoma | C77-90 |
| HIV | B20, B21, B22, B24 |
| Hypertension | I10-I16 |
| Previous stroke, TIA, or embolism | I63, I64, I679, I693, I694, I698, I67, I69, Z866, Z876, G453, G458, G459, I74 |
| Anaemia | D50-59, D60-64 |
| Alcoholism | E244, F10, G312, G621, G721, I426, K292, K70, K860, O354, P043, Q860, T51, Y90, Y91, Y91, Z502, Z714 |
| Prior bleed | I60, I61, I62, S064, S065, S066, I850, I983, K25-28 (subcodes 0-2 and 4-6 only), K625, K922, D62 |
| **Medication** | **ATC code beginning with** |
| Warfarin | B01AA03 |
| NOAC | B01AF02, B01AE07, B01AF03, B01AF01 |
| Antiplatelet | B01AC06, B01AC04, B01AC24, B01AC22 |
| Diuretic | C03A, C03B, C03C, C03D, C03E |
| Beta blocker | C07A, C07B, C07C, C07D, C07E, C07F |
| Ca channel blocker | C08C, C08D, C08E, C08G |
| RAAS inhibitor | C09A, C09B, C09C, C09D, C09X |
| Statin | C10AA |
| Oral antidiabetic drug | A10B |
| Insulin | A10A |
| Antidepressant | N06A |
| Digoxin | C01AA05 |
| Rhythm control drug | C01B, C07AA07 |
| Corticosteroids | H02A |
| PPI | A02BC |

**eTable 2a: Complete baseline characteristics ischemic stroke cohort.**

| **Baseline characteristics of ischaemic stroke cohort** | **NOAC (N=454)** | **Warfarin (N=1229)** | **Antiplatelet (N=2026)** | **No treatment (N=2308)** |
| --- | --- | --- | --- | --- |
| Female sex, n (%) | 237 (52.2%) | 577 (46.9%) | 1149 (56.7%) | 1238 (53.6%) |
| Low dose NOAC | 218 (48.0%) | NA | NA | NA |
| Mean duration (years (SD))** | 1.2 (1.2) | 2.9 (2.0) | 2.7 (1.8) | 0.8 (1.0) |
| Age | | | | |
| Mean (sd) | 79.25 (9.35) | 80.62 (8.45) | 83.89 (9.24) | 80.64 (10.60) |
| 0-65 | 27 (5.9%) | 58 (4.7%) | 82 (4.0%) | 207 (9.0%) |
| 66-75 | 110 (24.2%) | 228 (18.6%) | 261 (12.9%) | 403 (17.5%) |
| 76-85 | 180 (39.6%) | 529 (43.0%) | 571 (28.2%) | 760 (32.9%) |
| 86-95 | 132 (29.1%) | 392 (31.9%) | 992 (49.0%) | 845 (36.6%) |
| 95+ | 5 (1.1%) | 22 (1.8%) | 120 (5.9%) | 93 (4.0%) |
| Charlson Comorbidity Index | | | | |
| Mean (sd) | 5.59 (2.35) | 5.92 (2.40) | 6.18 (2.39) | 5.75 (2.59) |
| 0-2 | 24 (5.3%) | 40 (3.3%) | 63 (3.1%) | 172 (7.5%) |
| 3-4 | 137 (30.2%) | 324 (26.4%) | 398 (19.6%) | 596 (25.8%) |
| 4+ | 293 (64.5%) | 865 (70.4%) | 1565 (77.2%) | 1540 (66.7%) |
| CHA_2_DS_2_-VASc | | | | |
| Mean (sd) | 4.43 (1.68) | 4.66 (1.64) | 4.80 (1.69) | 4.23 (1.84) |
| 0-1 | 15 (3.3%) | 24 (2.0%) | 50 (2.5%) | 171 (7.4%) |
| 2-3 | 117 (25.8%) | 276 (22.5%) | 388 (19.2%) | 617 (26.7%) |
| 3+ | 322 (70.9%) | 929 (75.6%) | 1588 (78.4%) | 1520 (65.9%) |
| HAS-BLED | | | | |
| Mean (sd) | 2.32 (0.88) | 2.29 (0.84) | 2.36 (0.95) | 2.25 (1.06) |
| 0-3 | 414 (91.2%) | 1132 (92.1%) | 1802 (88.9%) | 2035 (88.2%) |
| 3+ | 40 (8.8%) | 97 (7.9%) | 224 (11.1%) | 273 (11.8%) |
| Comorbidities underlying scores, n (%) | | | | |
| Myocardial infarction | 56 (12.3%) | 207 (16.8%) | 403 (19.9%) | 283 (12.3%) |
| Heart failure | 153 (33.7%) | 531 (43.2%) | 805 (39.7%) | 825 (35.7%) |
| Peripheral vascular disease | 57 (12.6%) | 150 (12.2%) | 250 (12.3%) | 240 (10.4%) |
| Cerebral vascular disease | 135 (29.7%) | 369 (30.0%) | 678 (33.5%) | 637 (27.6%) |
| Dementia | 39 (8.6%) | 69 (5.6%) | 319 (15.7%) | 251 (10.9%) |
| COPD | 67 (14.8%) | 233 (19.0%) | 328 (16.2%) | 350 (15.2%) |
| Peptic ulcer | 17 (3.7%) | 33 (2.7%) | 71 (3.5%) | 101 (4.4%) |
| Rheumatoid arthritis | 27 (5.9%) | 86 (7.0%) | 122 (6.0%) | 154 (6.7%) |
| Mild liver disease | 4 (0.9%) | 13 (1.1%) | 35 (1.7%) | 61 (2.6%) |
| Uncomplicated diabetes | 98 (21.6%) | 300 (24.4%) | 433 (21.4%) | 437 (18.9%) |
| Connective tissue disease | 13 (2.9%) | 17 (1.4%) | 48 (2.4%) | 54 (2.3%) |
| Renal disease | 43 (9.5%) | 130 (10.6%) | 216 (10.7%) | 257 (11.1%) |
| Complicated diabetes | 37 (8.1%) | 84 (6.8%) | 142 (7.0%) | 167 (7.2%) |
| Cancer | 66 (14.5%) | 195 (15.9%) | 320 (15.8%) | 378 (16.4%) |
| Moderate to severe liver disease | 2 (0.4%) | 5 (0.4%) | 11 (0.5%) | 14 (0.6%) |
| Metastatic carcinoma | 8 (1.8%) | 30 (2.4%) | 41 (2.0%) | 54 (2.3%) |
| HIV | 0 (0.0%) | 0 (0.0%) | 2 (0.1%) | 2 (0.1%) |
| Hypertension | 357 (78.6%) | 973 (79.2%) | 1540 (76.0%) | 1595 (69.1%) |
| Previous stroke, TIA, or embolism | 141 (31.1%) | 383 (31.2%) | 672 (33.2%) | 605 (26.2%) |
| Anaemia | 82 (18.1%) | 220 (17.9%) | 404 (19.9%) | 531 (23.0%) |
| Alcoholism | 13 (2.9%) | 22 (1.8%) | 77 (3.8%) | 149 (6.5%) |
| Prior bleed | 54 (11.9%) | 100 (8.1%) | 237 (11.7%) | 353 (15.3%) |
| Comedication, n (%) | | | | |
| Concomitant antiplatelet* | 37 (8.1%) | 107 (8.7%) | 49 (2.4%) | NA |
| Diuretic | 185 (40.7%) | 597 (48.6%) | 968 (47.8%) | 868 (37.6%) |
| Beta blocker | 347 (76.4%) | 916 (74.5%) | 1325 (65.4%) | 1309 (56.7%) |
| Ca channel blocker | 131 (28.9%) | 324 (26.4%) | 444 (21.9%) | 446 (19.3%) |
| RAAS inhibitor | 242 (53.3%) | 702 (57.1%) | 899 (44.4%) | 873 (37.8%) |
| Statin | 158 (34.8%) | 473 (38.5%) | 568 (28.0%) | 468 (20.3%) |
| Oral antidiabetic drug | 47 (10.4%) | 128 (10.4%) | 161 (7.9%) | 157 (6.8%) |
| Insulin | 35 (7.7%) | 112 (9.1%) | 183 (9.0%) | 175 (7.6%) |
| Antidepressant | 86 (18.9%) | 160 (13.0%) | 323 (15.9%) | 334 (14.5%) |
| Digoxin | 68 (15.0%) | 249 (20.3%) | 305 (15.1%) | 281 (12.2%) |
| Rhythm control drug | 18 (4.0%) | 32 (2.6%) | 42 (2.1%) | 52 (2.3%) |
| NSAID | 23 (5.1%) | 47 (3.8%) | 115 (5.7%) | 121 (5.2%) |
| Corticosteroid | 42 (9.3%) | 113 (9.2%) | 173 (8.5%) | 179 (7.8%) |
| PPI | 130 (28.6%) | 280 (22.8%) | 596 (29.4%) | 552 (23.9%) |

** For the no treatment group, this is the mean number of years since a last prescription, only of patients that ever received any antithrombotic treatment

**eTable 2b: Complete baseline characteristics intracranial hemorrhage cohort.**

| **Baseline characteristics of intracranial haemorrhage cohort** | **NOAC (N=311)** | **Warfarin (N=1028)** | **Antiplatelet (N=595)** | **No treatment (N=1072)** |
| --- | --- | --- | --- | --- |
| Female sex, n (%) | 132 (42.4%) | 415 (40.4%) | 275 (46.2%) | 442 (41.2%) |
| Low dose NOAC | 136 (43.7%) | NA | NA | NA |
| Mean duration (years (SD))** | 1.4 (1.3) | 3.1 (2.1) | 2.9 (2.0) | 0.6 (0.7) |
| Age | | | | |
| Mean (sd) | 80.02 (9.12) | 79.62 (8.75) | 83.02 (9.32) | 79.32 (10.92) |
| 0-65 | 17 (5.5%) | 60 (5.8%) | 28 (4.7%) | 113 (10.5%) |
| 66-75 | 65 (20.9%) | 217 (21.1%) | 88 (14.8%) | 203 (18.9%) |
| 76-85 | 128 (41.2%) | 445 (43.3%) | 183 (30.8%) | 385 (35.9%) |
| 86-95 | 88 (28.3%) | 295 (28.7%) | 261 (43.9%) | 338 (31.5%) |
| 95+ | 13 (4.2%) | 11 (1.1%) | 35 (5.9%) | 33 (3.1%) |
| Charlson Comorbidity Index | | | | |
| Mean (sd) | 5.83 (2.53) | 5.80 (2.46) | 6.52 (2.58) | 5.93 (2.83) |
| 0-2 | 15 (4.8%) | 38 (3.7%) | 11 (1.8%) | 79 (7.4%) |
| 3-4 | 89 (28.6%) | 295 (28.7%) | 103 (17.3%) | 278 (25.9%) |
| 4+ | 207 (66.6%) | 695 (67.6%) | 481 (80.8%) | 715 (66.7%) |
| CHA_2_DS_2_-VASc | | | | |
| Mean (sd) | 4.33 (1.71) | 4.31 (1.64) | 4.76 (1.64) | 4.07 (1.83) |
| 0-1 | 14 (4.5%) | 30 (2.9%) | 13 (2.2%) | 86 (8.0%) |
| 2-3 | 83 (26.7%) | 308 (30.0%) | 119 (20.0%) | 330 (30.8%) |
| 3+ | 214 (68.8%) | 690 (67.1%) | 463 (77.8%) | 656 (61.2%) |
| HAS-BLED | | | | |
| Mean (sd) | 2.35 (0.91) | 2.26 (0.85) | 2.52 (0.98) | 2.36 (1.02) |
| 0-3 | 281 (90.4%) | 961 (93.5%) | 505 (84.9%) | 932 (86.9%) |
| 3+ | 30 (9.6%) | 67 (6.5%) | 90 (15.1%) | 140 (13.1%) |
| Comorbidities underlying scores, n (%) | | | | |
| Myocardial infarction | 32 (10.3%) | 140 (13.6%) | 128 (21.5%) | 141 (13.2%) |
| Heart failure | 103 (33.1%) | 421 (41.0%) | 247 (41.5%) | 358 (33.4%) |
| Peripheral vascular disease | 29 (9.3%) | 106 (10.3%) | 84 (14.1%) | 98 (9.1%) |
| Cerebral vascular disease | 91 (29.3%) | 285 (27.7%) | 221 (37.1%) | 339 (31.6%) |
| Dementia | 39 (12.5%) | 84 (8.2%) | 123 (20.7%) | 107 (10.0%) |
| COPD | 64 (20.6%) | 157 (15.3%) | 96 (16.1%) | 167 (15.6%) |
| Peptic ulcer | 9 (2.9%) | 32 (3.1%) | 26 (4.4%) | 48 (4.5%) |
| Rheumatoid arthritis | 20 (6.4%) | 62 (6.0%) | 35 (5.9%) | 54 (5.0%) |
| Mild liver disease | 4 (1.3%) | 15 (1.5%) | 9 (1.5%) | 50 (4.7%) |
| Uncomplicated diabetes | 78 (25.1%) | 222 (21.6%) | 132 (22.2%) | 205 (19.1%) |
| Connective tissue disease | 18 (5.8%) | 16 (1.6%) | 19 (3.2%) | 41 (3.8%) |
| Renal disease | 27 (8.7%) | 127 (12.4%) | 86 (14.5%) | 139 (13.0%) |
| Complicated diabetes | 21 (6.8%) | 71 (6.9%) | 43 (7.2%) | 66 (6.2%) |
| Cancer | 56 (18.0%) | 172 (16.7%) | 108 (18.2%) | 201 (18.8%) |
| Moderate to severe liver disease | 1 (0.3%) | 4 (0.4%) | 6 (1.0%) | 11 (1.0%) |
| Metastatic carcinoma | 9 (2.9%) | 26 (2.5%) | 20 (3.4%) | 52 (4.9%) |
| HIV | 0 (0.0%) | 0 (0.0%) | 0 (0.0%) | 1 (0.1%) |
| Hypertension | 244 (78.5%) | 769 (74.8%) | 453 (76.1%) | 751 (70.1%) |
| Previous stroke, TIA, or embolism | 91 (29.3%) | 289 (28.1%) | 208 (35.0%) | 291 (27.1%) |
| Anaemia | 64 (20.6%) | 193 (18.8%) | 165 (27.7%) | 272 (25.4%) |
| Alcoholism | 10 (3.2%) | 37 (3.6%) | 43 (7.2%) | 95 (8.9%) |
| Prior bleed | 47 (15.1%) | 94 (9.1%) | 105 (17.6%) | 224 (20.9%) |
| Comedication, n (%) | | | | |
| Concomitant antiplatelet* | 5 (1.6%) | 25 (2.4%) | 12 (2.0%) | NA |
| Diuretic | 129 (41.5%) | 454 (44.2%) | 293 (49.2%) | 399 (37.2%) |
| Beta blocker | 222 (71.4%) | 712 (69.3%) | 362 (60.8%) | 631 (58.9%) |
| Ca channel blocker | 81 (26.0%) | 248 (24.1%) | 128 (21.5%) | 197 (18.4%) |
| RAAS inhibitor | 165 (53.1%) | 566 (55.1%) | 268 (45.0%) | 424 (39.6%) |
| Statin | 110 (35.4%) | 399 (38.8%) | 200 (33.6%) | 279 (26.0%) |
| Oral antidiabetic drug | 34 (10.9%) | 101 (9.8%) | 45 (7.6%) | 75 (7.0%) |
| Insulin | 25 (8.0%) | 94 (9.1%) | 56 (9.4%) | 71 (6.6%) |
| Antidepressant | 69 (22.2%) | 166 (16.1%) | 149 (25.0%) | 187 (17.4%) |
| Digoxin | 48 (15.4%) | 171 (16.6%) | 80 (13.4%) | 124 (11.6%) |
| Rhythm control drug | 11 (3.5%) | 34 (3.3%) | 9 (1.5%) | 25 (2.3%) |
| NSAID | 20 (6.4%) | 37 (3.6%) | 34 (5.7%) | 42 (3.9%) |
| Corticosteroid | 26 (8.4%) | 91 (8.9%) | 50 (8.4%) | 100 (9.3%) |
| PPI | 90 (28.9%) | 228 (22.2%) | 178 (29.9%) | 276 (25.7%) |

** For the no treatment group, this is the mean number of years since a last prescription, only of patients that ever received any antithrombotic treatment

**eTable 2c: Complete baseline characteristics severe gastrointestinal bleed cohort**

| **Baseline table of severe gastrointestinal bleed cohort** | **NOAC (N=652)** | **Warfarin (N=1293)** | **Antiplatelet (N=893)** | **No treatment (N=1453)** |
| --- | --- | --- | --- | --- |
| Female sex, n (%) | 300 (46.0%) | 526 (40.7%) | 412 (46.1%) | 607 (41.8%) |
| Low dose NOAC | 254 (39.0%) | NA | NA | NA |
| Mean duration (years (SD))** | 1.3 (1.2) | 2.9 (2.1) | 3.0 (2.0) | 0.8 (1.0) |
| Age | | | | |
| Mean (sd) | 77.84 (9.36) | 78.39 (9.60) | 81.59 (10.40) | 77.68 (11.43) |
| 0-65 | 52 (8.0%) | 103 (8.0%) | 64 (7.2%) | 185 (12.7%) |
| 66-75 | 188 (28.8%) | 331 (25.6%) | 161 (18.0%) | 360 (24.8%) |
| 76-85 | 264 (40.5%) | 510 (39.4%) | 267 (29.9%) | 474 (32.6%) |
| 86-95 | 135 (20.7%) | 331 (25.6%) | 354 (39.6%) | 396 (27.3%) |
| 95+ | 13 (2.0%) | 18 (1.4%) | 47 (5.3%) | 38 (2.6%) |
| Charlson Comorbidity Index | | | | |
| Mean (sd) | 5.77 (2.63) | 6.09 (2.65) | 6.61 (2.68) | 6.29 (3.09) |
| 0-2 | 42 (6.4%) | 47 (3.6%) | 29 (3.2%) | 108 (7.4%) |
| 3-4 | 192 (29.4%) | 333 (25.8%) | 167 (18.7%) | 326 (22.4%) |
| 4+ | 418 (64.1%) | 913 (70.6%) | 697 (78.1%) | 1019 (70.1%) |
| CHADsVASc | | | | |
| Mean (sd) | 4.21 (1.81) | 4.26 (1.65) | 4.58 (1.74) | 3.93 (1.86) |
| 0-1 | 38 (5.8%) | 40 (3.1%) | 32 (3.6%) | 147 (10.1%) |
| 2-3 | 206 (31.6%) | 385 (29.8%) | 211 (23.6%) | 448 (30.8%) |
| 3+ | 408 (62.6%) | 868 (67.1%) | 650 (72.8%) | 858 (59.1%) |
| HAS-BLED | | | | |
| Mean (sd) | 2.25 (0.93) | 2.26 (0.92) | 2.41 (1.02) | 2.34 (1.17) |
| 0-3 | 599 (91.9%) | 1180 (91.3%) | 768 (86.0%) | 1233 (84.9%) |
| 3+ | 53 (8.1%) | 113 (8.7%) | 125 (14.0%) | 220 (15.1%) |
| Comorbidities underlying scores, n (%) | | | | |
| Myocardial infarction | 84 (12.9%) | 220 (17.0%) | 242 (27.1%) | 202 (13.9%) |
| Heart failure | 259 (39.7%) | 623 (48.2%) | 397 (44.5%) | 567 (39.0%) |
| Peripheral vascular disease | 72 (11.0%) | 152 (11.8%) | 149 (16.7%) | 171 (11.8%) |
| Cerebral vascular disease | 160 (24.5%) | 244 (18.9%) | 229 (25.6%) | 314 (21.6%) |
| Dementia | 29 (4.4%) | 69 (5.3%) | 124 (13.9%) | 99 (6.8%) |
| COPD | 129 (19.8%) | 277 (21.4%) | 191 (21.4%) | 302 (20.8%) |
| Peptic ulcer | 26 (4.0%) | 79 (6.1%) | 73 (8.2%) | 144 (9.9%) |
| Rheumatoid arthritis | 41 (6.3%) | 119 (9.2%) | 54 (6.0%) | 107 (7.4%) |
| Mild liver disease | 19 (2.9%) | 39 (3.0%) | 32 (3.6%) | 91 (6.3%) |
| Uncomplicated diabetes | 159 (24.4%) | 339 (26.2%) | 226 (25.3%) | 328 (22.6%) |
| Connective tissue disease | 29 (4.4%) | 18 (1.4%) | 16 (1.8%) | 46 (3.2%) |
| Renal disease | 62 (9.5%) | 206 (15.9%) | 157 (17.6%) | 263 (18.1%) |
| Complicated diabetes | 64 (9.8%) | 106 (8.2%) | 92 (10.3%) | 131 (9.0%) |
| Cancer | 131 (20.1%) | 269 (20.8%) | 203 (22.7%) | 345 (23.7%) |
| Moderate to severe liver disease | 6 (0.9%) | 10 (0.8%) | 11 (1.2%) | 44 (3.0%) |
| Metastatic carcinoma | 17 (2.6%) | 46 (3.6%) | 24 (2.7%) | 83 (5.7%) |
| HIV | 0 (0.0%) | 0 (0.0%) | 0 (0.0%) | 1 (0.1%) |
| Hypertension | 499 (76.5%) | 999 (77.3%) | 664 (74.4%) | 988 (68.0%) |
| Previous stroke, TIA, or embolism | 158 (24.2%) | 257 (19.9%) | 228 (25.5%) | 301 (20.7%) |
| Anaemia | 179 (27.5%) | 413 (31.9%) | 339 (38.0%) | 578 (39.8%) |
| Alcoholism | 36 (5.5%) | 40 (3.1%) | 67 (7.5%) | 139 (9.6%) |
| Prior bleed | 76 (11.7%) | 154 (11.9%) | 146 (16.3%) | 276 (19.0%) |
| Comedication, n (%) | | | | |
| Concomitant antiplatelet* | 41 (7.8%) | 129 (8.1%) | 54 (2.5%) | NA |
| Diuretic | 289 (44.3%) | 711 (55.0%) | 479 (53.6%) | 624 (42.9%) |
| Beta blocker | 508 (77.9%) | 928 (71.8%) | 555 (62.2%) | 819 (56.4%) |
| Ca channel blocker | 167 (25.6%) | 311 (24.1%) | 206 (23.1%) | 276 (19.0%) |
| RAAS inhibitor | 358 (54.9%) | 740 (57.2%) | 433 (48.5%) | 556 (38.3%) |
| Statin | 238 (36.5%) | 479 (37.0%) | 322 (36.1%) | 339 (23.3%) |
| Oral antidiabetic drug | 71 (10.9%) | 152 (11.8%) | 61 (6.8%) | 105 (7.2%) |
| Insulin | 61 (9.4%) | 127 (9.8%) | 90 (10.1%) | 138 (9.5%) |
| Antidepressant | 123 (18.9%) | 163 (12.6%) | 159 (17.8%) | 240 (16.5%) |
| Digoxin | 88 (13.5%) | 204 (15.8%) | 87 (9.7%) | 145 (10.0%) |
| Rhythm control drug | 24 (3.7%) | 57 (4.4%) | 18 (2.0%) | 33 (2.3%) |
| NSAID | 51 (7.8%) | 67 (5.2%) | 84 (9.4%) | 112 (7.7%) |
| Corticosteroid | 71 (10.9%) | 188 (14.5%) | 95 (10.6%) | 192 (13.2%) |
| PPI | 234 (35.9%) | 414 (32.0%) | 361 (40.4%) | 527 (36.3%) |

** For the no treatment group, this is the mean number of years since a last prescription, only of patients that ever received any antithrombotic treatment

**eTable 3a: Baseline characteristics and standardized mean differences of the propensity score matched cohort after ischemic stroke.**

|  | **NOAC** | **Warf** | **SMD** | **NOAC** | **AP** | **SMD** | **NOAC** | **None** | **SMD** |
| --- | --- | --- | --- | --- | --- | --- | --- | --- | --- |
| Age | 79,75 | 80,13 | -0,04 | 80,42 | 80,67 | -0,03 | 79,26 | 79,53 | -0,03 |
| Female sex | 52% | 51% | 0,02 | 53% | 52% | 0,03 | 52% | 55% | -0,05 |
| MI | 13% | 14% | -0,06 | 13% | 15% | -0,05 | 12% | 12% | 0,01 |
| CHF | 36% | 37% | -0,02 | 33% | 36% | -0,05 | 34% | 35% | -0,00 |
| Peripheral vascular disease | 12% | 13% | -0,03 | 11% | 12% | -0,01 | 12% | 10% | 0,08 |
| cerebral vascular disease | 29% | 28% | 0,03 | 30% | 32% | -0,05 | 30% | 29% | 0,02 |
| dementia | 9% | 7% | 0,05 | 10% | 10% | -0,01 | 9% | 8% | 0,02 |
| COPD | 15% | 17% | -0,06 | 14% | 15% | -0,01 | 15% | 15% | -0,01 |
| Peptic ulcer | 3% | 3% | - | 4% | 3% | 0,01 | 4% | 4% | 0,01 |
| Rheumatoid arthritis | 6% | 6% | -0,01 | 6% | 5% | 0,05 | 6% | 6% | 0,01 |
| Mild liver disease | 1% | 0% | 0,05 | 1% | 1% | -0,03 | 1% | 0% | 0,05 |
| Diabetes without complications | 21% | 23% | -0,04 | 22% | 24% | -0,04 | 22% | 22% | -0,01 |
| Connective tissue damage | 3% | 2% | 0,04 | 3% | 3% | 0,02 | 3% | 3% | 0,01 |
| Renal disease | 10% | 9% | 0,02 | 10% | 9% | 0,03 | 10% | 10% | -0,03 |
| Diabetes with complications | 8% | 9% | -0,03 | 9% | 9% | -0,01 | 8% | 9% | -0,02 |
| Cancer | 15% | 14% | 0,03 | 14% | 13% | 0,04 | 15% | 14% | 0,01 |
| Metastatic carcinoma | 2% | 1% | 0,02 | 2% | 2% | - | 2% | 2% | -0,02 |
| Hypertension | 79% | 80% | -0,02 | 80% | 80% | -0,01 | 79% | 78% | 0,02 |
| Stroke/TIA/Embolism | 31% | 30% | 0,01 | 31% | 33% | -0,04 | 31% | 29% | 0,04 |
| Anaemia | 18% | 18% | 0,01 | 19% | 19% | -0,01 | 18% | 19% | -0,01 |
| Alcoholism | 2% | 2% | - | 3% | 3% | - | 3% | 2% | 0,03 |
| Prior bleed | 11% | 11% | 0,01 | 12% | 11% | 0,02 | 12% | 15% | -0,09 |
| Diuretic | 42% | 44% | -0,04 | 41% | 40% | 0,02 | 41% | 41% | -0,00 |
| Beta blocker | 77% | 76% | 0,01 | 75% | 75% | 0,01 | 76% | 76% | 0,02 |
| Ca channel blocker | 28% | 31% | -0,05 | 29% | 29% | - | 29% | 27% | 0,04 |
| RAAS inhibitor | 53% | 53% | 0,00 | 52% | 53% | -0,01 | 53% | 49% | 0,09 |
| Statin | 35% | 38% | -0,05 | 34% | 34% | - | 34% | 32% | 0,06 |
| Oral antidiabetic | 10% | 10% | -0,01 | 11% | 11% | -0,01 | 10% | 10% | 0,01 |
| Insulin | 8% | 8% | -0,03 | 8% | 10% | -0,08 | 8% | 9% | -0,04 |
| Antidepressants | 19% | 19% | - | 18% | 19% | -0,02 | 19% | 20% | -0,02 |
| Digoxin | 15% | 15% | 0,01 | 14% | 14% | - | 15% | 15% | 0,01 |
| Rhythm control drugs | 3% | 3% | 0,01 | 2% | 2% | 0,01 | 4% | 3% | 0,02 |
| NSAIDs | 5% | 4% | 0,01 | 5% | 4% | 0,07 | 5% | 5% | 0,01 |
| corticosteroids | 9% | 9% | 0,01 | 9% | 7% | 0,07 | 9% | 7% | 0,06 |
| PPI | 28% | 26% | 0,06 | 28% | 31% | -0,06 | 29% | 27% | 0,04 |
| Year 2011 | 0% | 0% | - | 0% | 1% | -0,11 | 0% | 0% | - |
| Year 2012 | 2% | 2% | -0,04 | 2% | 2% | -0,02 | 2% | 1% | 0,05 |
| Year 2013 | 4% | 3% | 0,04 | 4% | 3% | 0,05 | 4% | 3% | 0,06 |
| Year 2014 | 11% | 14% | -0,09 | 12% | 13% | -0,03 | 10% | 10% | - |
| Year 2015 | 18% | 20% | -0,04 | 18% | 22% | -0,11 | 17% | 17% | 0,02 |
| Year 2016 | 27% | 27% | -0,01 | 26% | 28% | -0,03 | 26% | 28% | -0,05 |
| Year 2017 | 28% | 26% | 0,06 | 28% | 24% | 0,08 | 30% | 32% | -0,05 |
| Year 2018 | 9% | 8% | 0,05 | 10% | 8% | 0,08 | 11% | 9% | 0,06 |

**eTable 3b: Baseline characteristics and standardized mean differences of the propensity score matched cohort after intracranial hemorrhage.**

|  | **NOAC** | **Warf** | **SMD** | **NOAC** | **AP** | **SMD** | **NOAC** | **None** | **SMD** |
| --- | --- | --- | --- | --- | --- | --- | --- | --- | --- |
| Age | 80,11 | 80,49 | -0,04 | 81,53 | 81,76 | -0,03 | 80,09 | 80,73 | -0,07 |
| Female sex | 43% | 42% | 0,01 | 47% | 47% | - | 43% | 41% | 0,03 |
| MI | 11% | 12% | -0,05 | 14% | 13% | 0,02 | 10% | 8% | 0,07 |
| CHF | 34% | 34% | 0,01 | 35% | 32% | 0,07 | 34% | 34% | 0,01 |
| Peripheral vascular disease | 9% | 8% | 0,02 | 10% | 10% | - | 9% | 8% | 0,03 |
| cerebral vascular disease | 28% | 28% | -0,01 | 35% | 35% | 0,01 | 30% | 30% | -0,09 |
| dementia | 12% | 11% | 0,03 | 15% | 17% | -0,05 | 13% | 11% | 0,04 |
| COPD | 21% | 19% | 0,05 | 16% | 14% | 0,06 | 21% | 18% | 0,06 |
| Peptic ulcer | 2% | 4% | -0,09 | 3% | 3% | -0,03 | 3% | 2% | 0,04 |
| Rheumatoid arthritis | 6% | 8% | -0,07 | 6% | 7% | -0,02 | 7% | 7% | -0,01 |
| Mild liver disease | 1% | 2% | -0,06 | 2% | 2% | -0,05 | 1% | 1% | - |
| Diabetes without complications | 25% | 25% | - | 25% | 23% | 0,04 | 24% | 22% | 0,05 |
| Connective tissue damage | 5% | 4% | 0,06 | 5% | 5% | - | 6% | 4% | 0,07 |
| Renal disease | 9% | 10% | -0,03 | 10% | 10% | - | 9% | 9% | -0,01 |
| Diabetes with complications | 7% | 8% | -0,03 | 6% | 8% | -0,09 | 7% | 6% | 0,05 |
| Cancer | 18% | 18% | 0,02 | 16% | 15% | 0,03 | 18% | 19% | -0,03 |
| Metastatic carcinoma | 3% | 3% | 0,02 | 1% | 1% | - | 3% | 3% | 0,02 |
| Hypertension | 77% | 76% | 0,03 | 79% | 77% | 0,06 | 78% | 80% | -0,04 |
| Stroke/TIA/Embolism | 28% | 27% | 0,03 | 35% | 35% | - | 30% | 30% | - |
| Anaemia | 20% | 20% | 0,01 | 23% | 24% | -0,01 | 21% | 20% | 0,02 |
| Alcoholism | 4% | 4% | -0,02 | 5% | 5% | - | 3% | 2% | 0,07 |
| Prior bleed | 11% | 11% | 0,01 | 15% | 18% | -0,08 | 15% | 14% | 0,02 |
| Diuretic | 43% | 43% | - | 42% | 42% | 0,01 | 41% | 41% | - |
| Beta blocker | 70% | 71% | -0,02 | 65% | 65% | - | 71% | 69% | 0,04 |
| Ca channel blocker | 26% | 26% | -0,01 | 26% | 22% | 0,08 | 26% | 29% | -0,07 |
| RAAS inhibitor | 54% | 54% | - | 47% | 48% | -0,01 | 52% | 50% | 0,06 |
| Statin | 36% | 33% | 0,06 | 37% | 39% | -0,05 | 35% | 33% | 0,05 |
| Oral antidiabetic | 11% | 11% | - | 10% | 11% | -0,04 | 10% | 10% | - |
| Insulin | 8% | 10% | -0,08 | 8% | 9% | -0,04 | 8% | 7% | 0,02 |
| Antidepressants | 21% | 20% | 0,01 | 27% | 23% | 0,08 | 22% | 20% | 0,05 |
| Digoxin | 16% | 16% | - | 15% | 15% | - | 15% | 13% | 0,07 |
| Rhythm control drugs | 4% | 4% | -0,02 | 2% | 4% | -0,09 | 4% | 3% | 0,04 |
| NSAIDs | 6% | 5% | 0,03 | 6% | 6% | 0,02 | 6% | 6% | - |
| corticosteroids | 9% | 11% | -0,09 | 10% | 9% | 0,04 | 8% | 8% | 0,01 |
| PPI | 27% | 27% | - | 33% | 31% | 0,05 | 29% | 29% | 0,01 |
| Year 2011 | 0% | 0% | - | 0% | 1% | - | 0% | 0% | - |
| Year 2012 | 2% | 1% | 0,06 | 3% | 3% | -0,05 | 2% | 2% | -0,03 |
| Year 2013 | 4% | 5% | -0,10 | 6% | 8% | -0,10 | 3% | 3% | - |
| Year 2014 | 7% | 7% | - | 10% | 12% | -0,05 | 6% | 5% | 0,05 |
| Year 2015 | 20% | 21% | -0,02 | 22% | 24% | -0,05 | 19% | 22% | -0,09 |
| Year 2016 | 21% | 20% | 0,03 | 21% | 22% | -0,01 | 20% | 20% | 0,02 |
| Year 2017 | 30% | 31% | -0,02 | 27% | 23% | 0,09 | 32% | 33% | -0,01 |
| Year 2018 | 16% | 14% | 0,05 | 10% | 8% | 0,08 | 18% | 16% | 0,06 |

**eTable 3c: Baseline characteristics and standardized mean differences of the propensity score matched cohort after severe gastrointestinal bleed.**

|  | **NOAC** | **Warf** | **SMD** | **NOAC** | **AP** | **SMD** | **NOAC** | **None** | **SMD** |
| --- | --- | --- | --- | --- | --- | --- | --- | --- | --- |
| Age | 77,97 | 78,21 | -0,03 | 79,18 | 79,88 | -0,07 | 77,86 | 77,96 | -0,01 |
| Female sex | 44% | 42% | 0,03 | 44% | 47% | -0,05 | 46% | 44% | 0,03 |
| MI | 14% | 15% | -0,03 | 18% | 21% | -0,09 | 13% | 14% | -0,02 |
| CHF | 45% | 46% | -0,03 | 42% | 43% | -0,02 | 40% | 40% | 0,01 |
| Peripheral vascular disease | 11% | 12% | -0,01 | 15% | 15% | -0,02 | 12% | 11% | 0,01 |
| cerebral vascular disease | 23% | 21% | 0,05 | 26% | 26% | -0,02 | 25% | 23% | 0,03 |
| dementia | 5% | 5% | -0,02 | 7% | 9% | -0,09 | 6% | 5% | 0,01 |
| COPD | 20% | 20% | -0,00 | 20% | 21% | -0,03 | 20% | 20% | -0,00 |
| Peptic ulcer | 5% | 6% | -0,04 | 5% | 5% | 0,01 | 5% | 5% | -0,02 |
| Rheumatoid arthritis | 6% | 8% | -0,04 | 6% | 6% | -0,02 | 7% | 7% | -0,01 |
| Mild liver disease | 3% | 3% | -0,04 | 4% | 5% | -0,06 | 3% | 2% | 0,05 |
| Diabetes without complications | 26% | 27% | -0,04 | 24% | 23% | 0,01 | 24% | 23% | 0,03 |
| Connective tissue damage | 4% | 2% | 0,08 | 4% | 3% | 0,06 | 5% | 4% | 0,02 |
| Renal disease | 12% | 12% | -0,01 | 12% | 14% | -0,09 | 11% | 11% | - |
| Diabetes with complications | 9% | 10% | -0,01 | 9% | 10% | -0,03 | 10% | 9% | 0,02 |
| Cancer | 22% | 23% | -0,04 | 21% | 21% | -0,01 | 21% | 21% | 0,01 |
| Metastatic carcinoma | 3% | 4% | -0,06 | 3% | 3% | -0,03 | 3% | 3% | - |
| Hypertension | 77% | 79% | -0,05 | 76% | 77% | -0,02 | 76% | 75% | 0,03 |
| Stroke/TIA/Embolism | 23% | 22% | 0,05 | 26% | 26% | -0,02 | 25% | 24% | 0,03 |
| Anaemia | 30% | 32% | -0,04 | 33% | 35% | -0,05 | 32% | 35% | -0,05 |
| Alcoholism | 4% | 5% | -0,02 | 7% | 7% | 0,01 | 6% | 7% | -0,06 |
| Prior bleed | 12% | 14% | -0,04 | 15% | 14% | 0,02 | 13% | 14% | -0,04 |
| Diuretic | 48% | 50% | -0,04 | 48% | 49% | -0,02 | 44% | 44% | - |
| Beta blocker | 78% | 77% | 0,01 | 73% | 72% | 0,04 | 76% | 74% | 0,05 |
| Ca channel blocker | 25% | 25% | -0,02 | 25% | 26% | -0,02 | 25% | 23% | 0,06 |
| RAAS inhibitor | 55% | 56% | -0,01 | 54% | 52% | 0,04 | 53% | 50% | 0,06 |
| Statin | 36% | 36% | 0,01 | 40% | 37% | 0,06 | 36% | 32% | 0,08 |
| Oral antidiabetic | 11% | 12% | -0,02 | 9% | 8% | 0,03 | 11% | 10% | 0,02 |
| Insulin | 9% | 9% | -0,01 | 9% | 10% | -0,05 | 9% | 9% | 0,01 |
| Antidepressants | 17% | 16% | 0,03 | 17% | 18% | -0,05 | 19% | 19% | 0,02 |
| Digoxin | 14% | 14% | 0,01 | 11% | 9% | 0,07 | 13% | 12% | 0,02 |
| Rhythm control drugs | 4% | 4% | - | 3% | 3% | - | 4% | 3% | 0,03 |
| NSAIDs | 7% | 7% | 0,01 | 10% | 9% | 0,03 | 7% | 8% | -0,02 |
| corticosteroids | 11% | 10% | 0,01 | 10% | 10% | - | 11% | 12% | -0,05 |
| PPI | 35% | 35% | -0,00 | 38% | 36% | 0,03 | 37% | 36% | 0,02 |
| Year 2011 | 0% | 0% | - | 0% | 1% | - | 0% | 1% | - |
| Year 2012 | 3% | 3% | -0,01 | 4% | 4% | -0,04 | 2% | 2% | 0,02 |
| Year 2013 | 4% | 5% | -0,02 | 6% | 6% | 0,01 | 4% | 3% | 0,04 |
| Year 2014 | 10% | 11% | -0,03 | 13% | 15% | -0,09 | 9% | 11% | -0,06 |
| Year 2015 | 22% | 21% | 0,03 | 23% | 24% | -0,03 | 20% | 20% | 0,00 |
| Year 2016 | 20% | 24% | -0,10 | 22% | 21% | 0,03 | 20% | 24% | -0,09 |
| Year 2017 | 27% | 26% | 0,01 | 20% | 19% | 0,02 | 27% | 25% | 0,03 |
| Year 2018 | 14% | 11% | 0,09 | 12% | 9% | 0,06 | 17% | 14% | 0,07 |

**eTable 4: Array approach sensitivity analyses for unmeasured confounder.**

| **Low HR** | **High HR** | **RR_CD_** | **P_C1_** | **P_C0_** | **Low HR adjusted** | **High HR adjusted** |
| --- | --- | --- | --- | --- | --- | --- |
| 1,36 | 1,57 | 1,0 | 0,50 | 0,1 | 1,36 | 1,57 |
| 1,36 | 1,57 | 1,5 | 0,50 | 0,1 | 1,14 | 1,32 |
| 1,36 | 1,57 | 2,0 | 0,50 | 0,1 | 1,00 | 1,15 |
| 1,36 | 1,57 | 2,5 | 0,50 | 0,1 | 0,89 | 1,03 |
| 1,36 | 1,57 | 3,0 | 0,50 | 0,1 | 0,82 | 0,94 |
| 1,36 | 1,57 | 3,5 | 0,50 | 0,1 | 0,76 | 0,87 |
| 1,36 | 1,57 | 4,0 | 0,50 | 0,1 | 0,71 | 0,82 |
| 1,36 | 1,57 | 4,5 | 0,50 | 0,1 | 0,67 | 0,77 |
| 1,36 | 1,57 | 5,0 | 0,50 | 0,1 | 0,63 | 0,73 |
| 1,36 | 1,57 | 1,0 | 0,40 | 0,1 | 1,36 | 1,57 |
| 1,36 | 1,57 | 1,5 | 0,40 | 0,1 | 1,19 | 1,37 |
| 1,36 | 1,57 | 2,0 | 0,40 | 0,1 | 1,07 | 1,23 |
| 1,36 | 1,57 | 2,5 | 0,40 | 0,1 | 0,98 | 1,13 |
| 1,36 | 1,57 | 3,0 | 0,40 | 0,1 | 0,91 | 1,05 |
| 1,36 | 1,57 | 3,5 | 0,40 | 0,1 | 0,85 | 0,98 |
| 1,36 | 1,57 | 4,0 | 0,40 | 0,1 | 0,80 | 0,93 |
| 1,36 | 1,57 | 4,5 | 0,40 | 0,1 | 0,77 | 0,88 |
| 1,36 | 1,57 | 5,0 | 0,40 | 0,1 | 0,73 | 0,85 |
| 1,36 | 1,57 | 1,0 | 0,30 | 0,1 | 1,36 | 1,57 |
| 1,36 | 1,57 | 1,5 | 0,30 | 0,1 | 1,24 | 1,43 |
| 1,36 | 1,57 | 2,0 | 0,30 | 0,1 | 1,15 | 1,33 |
| 1,36 | 1,57 | 2,5 | 0,30 | 0,1 | 1,08 | 1,25 |
| 1,36 | 1,57 | 3,0 | 0,30 | 0,1 | 1,02 | 1,18 |
| 1,36 | 1,57 | 3,5 | 0,30 | 0,1 | 0,97 | 1,12 |
| 1,36 | 1,57 | 4,0 | 0,30 | 0,1 | 0,93 | 1,07 |
| 1,36 | 1,57 | 4,5 | 0,30 | 0,1 | 0,90 | 1,03 |
| 1,36 | 1,57 | 5,0 | 0,30 | 0,1 | 0,87 | 1,00 |
| 1,36 | 1,57 | 1,0 | 0,20 | 0,1 | 1,36 | 1,57 |
| 1,36 | 1,57 | 1,5 | 0,20 | 0,1 | 1,30 | 1,50 |
| 1,36 | 1,57 | 2,0 | 0,20 | 0,1 | 1,25 | 1,44 |
| 1,36 | 1,57 | 2,5 | 0,20 | 0,1 | 1,20 | 1,39 |
| 1,36 | 1,57 | 3,0 | 0,20 | 0,1 | 1,17 | 1,35 |
| 1,36 | 1,57 | 3,5 | 0,20 | 0,1 | 1,13 | 1,31 |
| 1,36 | 1,57 | 4,0 | 0,20 | 0,1 | 1,11 | 1,28 |
| 1,36 | 1,57 | 4,5 | 0,20 | 0,1 | 1,1 | 1,25 |
| 1,36 | 1,57 | 5,0 | 0,20 | 0,1 | 1,1 | 1,22 |

**Low HR**: The weakest significant association found in the Cox regression

**High HR**: The strongest significant association found in the Cox regression

**RRcd**: The association of the confounder with mortality. E.g., if the RRcd is 3.0, it means a patient with this confounder is 3 times more likely to die

**Pc1**: The proportion of patients having the confounder in the comparator group

**Pc0**: The proportion of patients having the confounder in the NOAC group

**Low HR adjusted**: The HR of the weakest association if we take a confounder into account that has the properties of the columns on the left

**High HR adjusted**: The HR of the strongest association if we take a confounder into account that has the properties of the columns on the left

**eTable 5: Results of sensitivity analysis with different exposure definition.**

|  | NOAC | Warfarin | Antiplatelet | No treatment |
| --- | --- | --- | --- | --- |
| Ischaemic stroke | | | | |
| n main analysis (%) | 454 (7.5%) | 1229 (20.4%) | 2026 (33.7%) | 2308 (38.4%) |
| n sensitivity (%) | 577 (9.6%) | 1717 (28.5%) | 2290 (38.1%) | 1433 (23.8%) |
| 90 day mortality main analysis (%) | 17.6% | 17.6% | 29.8% | 26.3% |
| 90 day mortality sensitivity (%) | 18.2% | 19.3% | 30.7% | 25.8% |
| Intracranial haemorrhage | | | | |
| n main analysis (%) | 311 (10.3%) | 1028 (34.2%) | 595 (19.8%) | 1072 (35.7%) |
| n sensitivity (%) | 370 (12.3%) | 1377 (45.8%) | 675 (22.5%) | 584 (19.4%) |
| 90 day mortality main analysis (%) | 26.4% | 32.4% | 37.0% | 29.4% |
| 90 day mortality sensitivity (%) | 25.1% | 32.2% | 36.7% | 28.4% |
| Severe gastrointestinal bleed | | | | |
| n main analysis (%) | 652 (15.2%) | 1293 (30.1%) | 893 (20.8%) | 1453 (33.9%) |
| n sensitivity (%) | 765 (17.8%) | 1680 (39.2%) | 1014 (23.6%) | 832 (19.4%) |
| 90 day mortality main analysis (%) | 10.9% | 11.4% | 21.7% | 19.5% |
| 90 day mortality sensitivity (%) | 11.1% | 11.5% | 22.8% | 22.5% |

Results from the sensitivity analyses when any prescription in the 180 days prior to inclusion was used to assess treatment at the event. Proportion of patients treated with different antithrombotic treatments and 90 day mortality rates.

**eTable 6: Results of sensitivity analysis only including primary diagnoses from inpatient care.**

|  | NOAC | Warfarin | Antiplatelet | No treatment |
| --- | --- | --- | --- | --- |
| Intracranial haemorrhage | | | | |
| n main analysis (%) | 311 (10.3%) | 1028 (34.2%) | 595 (19.8%) | 1072 (35.7%) |
| n sensitivity (%) | 225 (10.0%) | 847 (37.8%) | 428 (19.1%) | 740 (33.0%) |
| 90 day mortality main analysis (%) | 26.4% | 32.4% | 37.0% | 29.4% |
| 90 day mortality sensitivity (%) | 26.7% | 32.7% | 39.5% | 31.5% |
| aHR sensitivity analysis | Reference | 1.37 (1.00 – 1.88) | 1.26 (0.87 – 1.84) | 1.00 (0.73 – 1.39) |
| Severe gastrointestinal bleed | | | | |
| n main analysis (%) | 652 (15.2%) | 1293 (30.1%) | 893 (20.8%) | 1453 (33.9%) |
| n sensitivity (%) | 271 (12.5%) | 681 (31.3%) | 513 (23.6%) | 709 (32.6%) |
| 90 day mortality main analysis (%) | 10.9% | 11.4% | 21.7% | 19.5% |
| 90 day mortality sensitivity (%) | 12.5% | 12.5% | 24.6% | 24.4% |
| aHR sensitivity analysis | Reference | 1.03 (0.66 – 1.63) | 1.82 (1.16 – 2.85) | 1.93 (1.28 – 2.92) |

Results from the sensitivity analyses when including only primary diagnosis from inpatient care. The aHR are adjusted hazard ratios from the cox regression with the same covariates as the main analysis.

**eTable 7: Results of sensitivity analysis excluding all patients with concomitant antiplatelet therapy**

|  | **NOAC** | **Warfarin** | **Antiplatelet** | **No treatment** |
| --- | --- | --- | --- | --- |
| Ischemic stroke | | | | |
| Original mortality | 80 (17.6%) | 216 (17.6%) | 604 (29.8%) | 608 (26.3%) |
| Mortality excluding double | 74 (17.7%) | 191 (17.0%) | 590 (29.8%) | 608 (26.3%) |
| Intracranial hemorrhage | | | | |
| Original mortality | 82 (26.4%) | 333 (32.4%) | 220 (37.0%) | 315 (29.4%) |
| Mortality excluding double | 77 (25.8%) | 308 (32.1%) | 208 (36.7%) | 315 (29.4%) |
| Severe gastrointestinal bleed | | | | |
| Original mortality | 71 (10.9%) | 147 (11.4%) | 194 (21.7%) | 284 (19.5%) |
| Mortality excluding double | 68 (11.4%) | 138 (12.0%) | 191 (22.7%) | 284 (19.5%) |

Results from the sensitivity analyses where all patients receiving concomitant antiplatelet therapy (i.e., NOAC + antiplatelet, warfarin + antiplatelet or double antiplatelet therapy) were excluded.

**eFigure 1a: Kaplan-Meier curves and p-values from the log-rank test in the trimmed propensity score matched cohorts after ischemic stroke.**

**
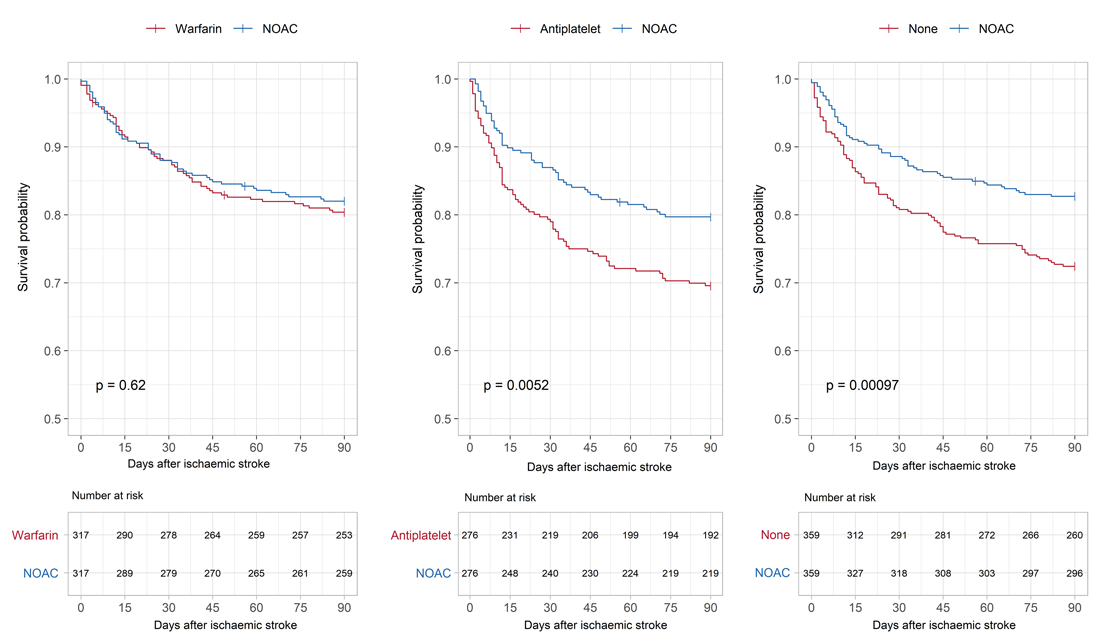
**

**eFigure 1b: Kaplan-Meier curves and p-values from the log-rank test in the trimmed propensity score matched cohorts after intracranial hemorrhage**

**
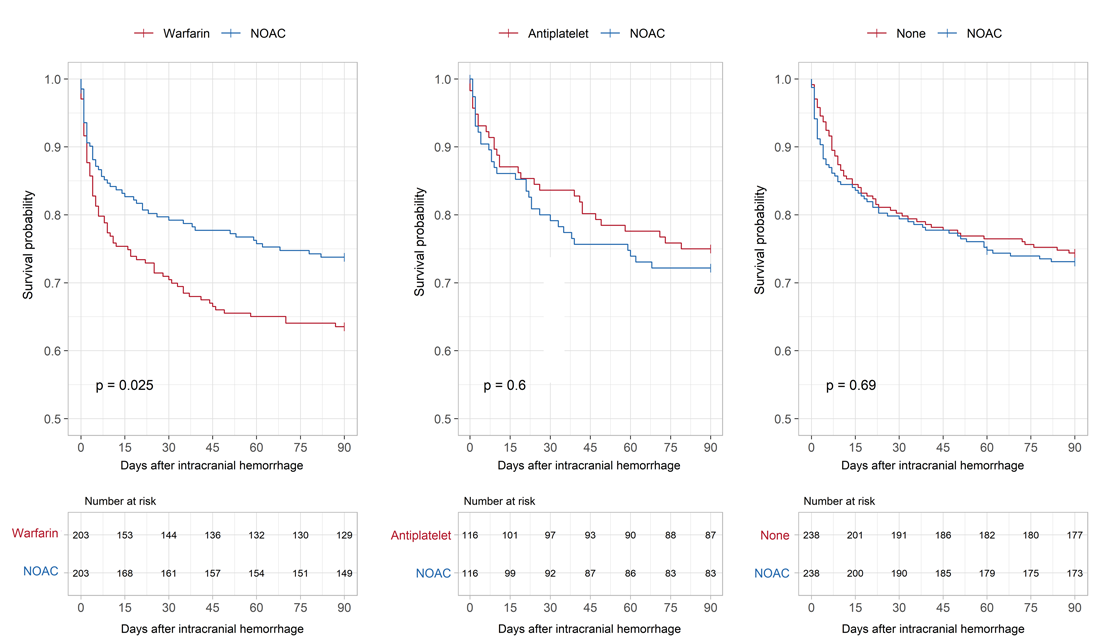
**

**eFigure 1c: Kaplan-Meier curves and p-values from the log rank test in the trimmed propensity score matched cohorts after severe gastrointestinal bleed.**

**
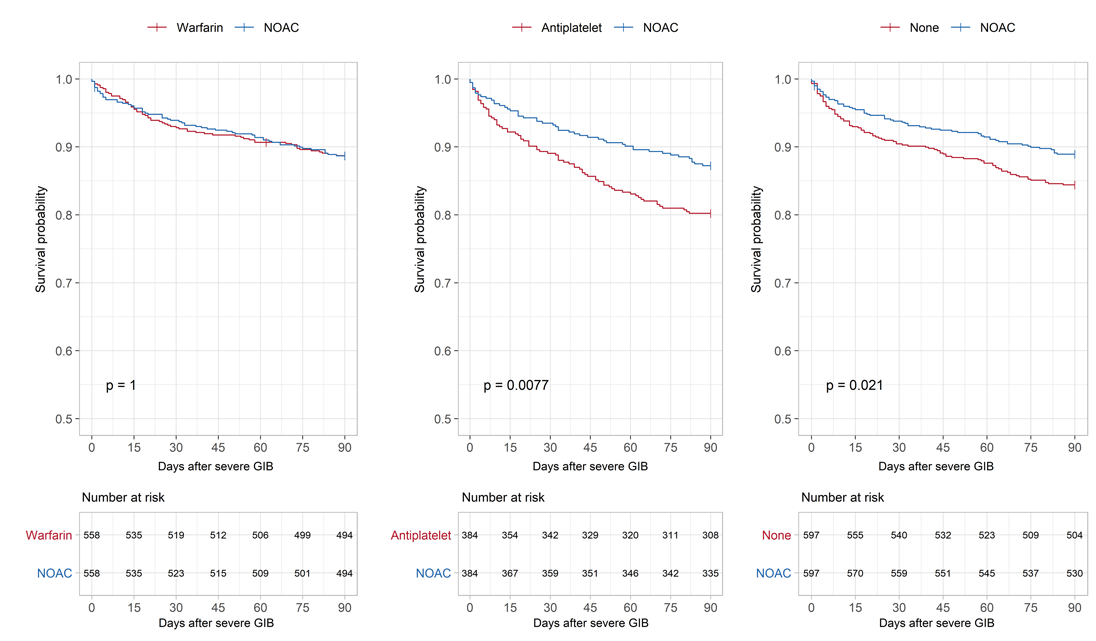
**
